# Subsegmental vascular dilatations: An important finding in COVID-19 pneumonia

**DOI:** 10.1590/0037-8682-0269-2026

**Published:** 2026-10-16

**Authors:** Gláucia Zanetti, Bruno Hochhegger, Alexandre Dias Mançano, Edson Marchiori

**Affiliations:** 1 Universidade Federal do Rio de Janeiro, Departamento de Radiologia, Rio de Janeiro, RJ, Brasil.; 2 University of Florida, Department of Radiology, Gainesville, FL, USA.

An 82-year-old man was admitted to the emergency department with a 5-day history of coryza, dry cough, and myalgia, which progressed to dyspnea and worsening cough. Laboratory test results were unremarkable. Chest computed tomography (CT) revealed ground-glass opacities with interspersed dilated vessels in the left lower lobe (Figure 1). Rapid tests for influenza A and B and respiratory syncytial virus were negative, whereas testing for coronavirus disease 2019 (COVID-19) was positive. The patient recovered uneventfully and was discharged after two weeks, asymptomatic, with normal CT findings (Figure 2).

**FIGURE 1:**
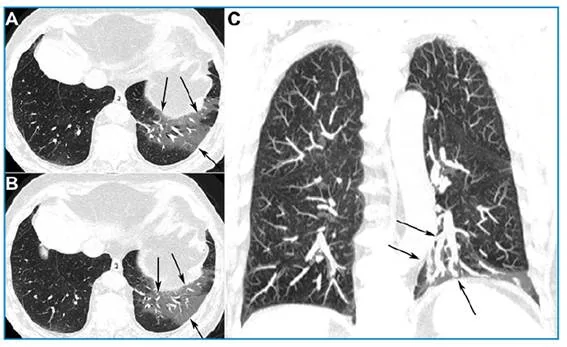
**(A,B)** Axial unenhanced chest CT scans showing dilated and tortuous vessels within a ground-glass opacity in the left lower lobe (arrows). **(C)** Coronal reconstruction showing the dilated segmental vessels (arrows). Note the normal vascular pattern in the same region of the right lung.

**FIGURE 2:**
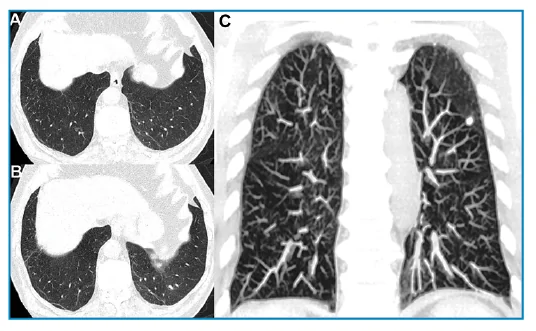
Axial unenhanced chest CT scans **(A and B)** and coronal reconstruction **(C)** performed two weeks after the first scan, showing a normal vascular pattern in the previously affected regions.

Although the peak of the COVID-19 pandemic has passed, a significant number of cases continue to occur. Computed tomography (CT) plays an important role in the evaluation of pulmonary infections. However, COVID-19 has nonspecific CT features, and differentiating it from other viral pneumonias can be challenging. Pulmonary vascular dilatations (PVDs) are an important tomographic finding that may help radiologists differentiate COVID-19 pneumonia from other viral pneumonias^1^ ^,^ ^2^.

PVDs observed in COVID-19 represent structural and functional vascular changes characterized by increased vascular caliber, resulting from complex endotheliopathy caused by several mechanisms. However, these mechanisms remain unclear, and their prevalence and distribution remain debated. Subsegmental vessel dilatations are highly prevalent in COVID-19 pneumonia and are not confined to lung opacities; they may also occur in a regional pattern outside these opacities^2^ ^-^ ^5^. In conclusion, the presence of PVDs on chest CT may help radiologists differentiate COVID-19 pneumonia from other viral pneumonias.
